# Parental obesity predisposes to exacerbated metabolic and inflammatory disturbances in childhood obesity within the framework of an altered profile of trace elements

**DOI:** 10.1038/s41387-024-00258-6

**Published:** 2024-01-18

**Authors:** Álvaro González-Domínguez, Lucía Jurado-Sumariva, Jesús Domínguez-Riscart, Ana Saez-Benito, Raúl González-Domínguez

**Affiliations:** 1 Instituto de Investigación e Innovación Biomédica de Cádiz (INiBICA), Hospital Universitario Puerta del Mar, Universidad de Cádiz, 11009 Cádiz, Spain; 2 Unidad de Endocrinología Pediátrica y Diabetes, Servicio de Pediatría, Hospital Universitario Puerta del Mar, 11009 Cádiz, Spain; 3 Unidad de Análisis Clínicos, Hospital Universitario Puerta del Mar, 11009 Cádiz, Spain

**Keywords:** Obesity, Metals

## Abstract

**Background:**

Family history of obesity is known to increase the odds of developing childhood obesity in the offspring, but its influence in underlying molecular complications remains unexplored.

**Subjects/Methods:**

Here, we investigated a population-based cohort comprising children with obesity, with and without parental obesity (PO+, *N* = 20; PO−, *N* = 29), and lean healthy children as controls (*N* = 30), from whom plasma and erythrocyte samples were collected to characterize their multi-elemental profile, inflammatory status, as well as carbohydrate and lipid metabolisms.

**Results:**

We found parental obesity to be associated with unhealthier outcomes in children, as reflected in increased blood insulin levels and reduced insulin sensitivity, unfavorable lipid profile, and pro-inflammatory milieu. This was accompanied by moderate alterations in the content of trace elements, including increased copper-to-zinc ratios and iron deficiency in circulation, as well as metal accumulation within erythrocytes.

**Conclusions:**

Therefore, we hypothesize that family history of obesity could be an important risk factor in modulating the characteristic multi-elemental alterations behind childhood obesity, which in turn could predispose to boost related comorbidities and metabolic complications.

## Introduction

In the last decades, obesity has become one of the most prevalent chronic diseases, reaching pandemic quotes [1]. Although the exact etiology is still unclear, it is well recognized that obesity is the result of an interplay between a complex set of risk factors, including diet, the environment, ethnicity, genetics, perinatal, and psychosocial factors [2, 3]. Among them, family history of obesity has repeatedly been associated with increased odds of developing obesity at early ages [4–8]. On the one hand, external stimuli during fetal development have been demonstrated to strongly influence the onset of childhood obesity, with prenatal exposure to maternal diseases (e.g., diabetes mellitus, high adiposity) and to inadequate maternal nutrition being linked to higher susceptibility of developing obesity in the infant [2, 9]. Although much less studied, paternal obesity may also induce programming mechanisms in the offspring (e.g., impairments in glucose and lipid metabolism, hormonal regulation), thereby resulting in elevated metabolic risk [10]. Furthermore, family history of obesity has been associated with lower methylation levels in differentially methylated regions of many genes, pointing to an epigenetic component in this parental predisposition to obesity [11]. Besides these genetic/biological determinants, it should be noted that parental obesity (PO) could also be “transmitted” to further generations through lifestyle habits, since children grown by families with obesity frequently have lower liking for vegetables and stronger preference for fatty foods, sedentary behaviors, and overeating-type eating styles [12].

As a result of this etiological complexity, childhood obesity is normally accompanied by a myriad of profound metabolic dysregulations, including disturbed homeostasis of insulin, carbohydrates, and lipids, chronic low-grade inflammation, and exacerbated oxidative stress [13]. In this respect, we have proven that children with obesity have increased blood levels of pro-inflammatory components and markers of lipid and protein oxidation, together with an impaired antioxidant system [14–16]. In particular, we found that blunted reducing power generation through the pentose phosphate pathway [14] and catalase post-translational modifications [15] could be plausible mechanisms behind these pathogenic events. Within this framework, growing evidence suggests that trace elements and heavy metals could act as modulators of these metabolic complications because of their participation in a multitude of essential biological processes. Recently, we described childhood obesity to be characterized by raised circulating levels of copper, decreased plasma content of various essential trace elements (i.e., Co, Cr, Mn, Mo, Se, Zn), and metal accumulation within erythrocytes [17]. Interestingly, this abnormal multi-elemental profile was found to be closely associated with the characteristic pathogenic events behind childhood obesity (i.e., hyperinsulinemia, dyslipidemia, inflammation, oxidative stress). Furthermore, we have demonstrated that these obesity-related metal perturbances can be modulated by various risk factors [18], such as diet [19], sex [20], pubertal development status [21], and insulin resistance severity [22]. However, the potential influence of PO in this disturbed metal profile in the offspring remains unexplored.

In the present work, we aimed to delve into the molecular pathogenic hallmarks that family history of obesity may imprint in the offspring with obesity. For this purpose, we recruited a population-based cohort comprising children with obesity, with and without PO, and healthy control children. Plasma and erythrocyte samples were collected to perform a comprehensive characterization of classical markers of insulin resistance, dyslipidemia, and inflammation, and to investigate their relationship with trace element status.

## Materials and methods

### Study population and sample collection

The study population consisted of prepubertal children (Tanner stage I, 6–12 years) with obesity, i.e., body mass index (BMI) over 2 standard deviations above the mean of the reference population [23]. Pediatric specialists registered data regarding PO at inclusion, which were employed to classify study participants as children with obesity and PO (PO+), when at least one of the parents had obesity (BMI > 30 kg/m^2^); or as children with obesity without PO (PO−), when none of the parents had obesity (BMI < 30 kg/m^2^). On the other hand, children without obesity nor PO needing a routine blood test were recruited as the control population. Family history of other comorbidities (i.e., type II diabetes, hypertension, dyslipidemia) was also registered from clinical records. The study participants did not receive any treatment (e.g., medication, lifestyle guidance) along the consecution of the research project. Using a sample size of 79 children, and considering an alpha risk of 0.05, the statistical power of our comparisons was above 80%, as calculated using the GRANMO 7.12 webtool.

Venous blood samples were collected in the morning after overnight fasting using serum and plasma separating tubes. Tubes were first centrifuged at 1500 × *g* for 10 min at 4 °C to separate serum and plasma samples. To obtain the erythrocyte fraction, the resulting pellets were then washed three times with cold saline solution (9 g/l NaCl, 4 °C) and centrifuged at 1500 × *g* for 5 min at 4 °C. The samples were finally aliquoted and stored at −80 °C until analysis. The study was performed in accordance with the principles contained in the Declaration of Helsinki. The Ethical Committee of “Hospital Universitario Puerta del Mar” (Cádiz, Spain) approved the study protocol (Ref. PI22/01899), and all participants and/or legal guardians provided written informed consent.

### Anthropometric and biochemical variables

Anthropometric data were evaluated by pediatric specialists, and *Z*-scores adjusted for age and gender were obtained using Spanish reference values [23]. An Alinity automatic analyzer (Abbot, Spain) was employed to quantify plasma insulin and glucose levels, lipid profile (i.e., total cholesterol; high-density lipoprotein cholesterol, HDL-C; low-density lipoprotein cholesterol, LDL-C; and triglycerides), glycated hemoglobin (HbA1c), C-reactive protein (CRP), iron metabolism-related variables (i.e., ferritin, transferrin, transferrin saturation, total iron binding capacity—TIBC), uric acid, and vitamins (i.e., folic acid, vitamin D, vitamin B12). The following formula were applied to calculate the homeostasis model assessment of insulin resistance (HOMA-IR) and Castelli risk index-I (CRI-I):

$${\rm{HOMA}}-{\rm{IR}}=\frac{{\rm{Glucose}}\,0\times {\rm{Insulin}}\,0\times 0.055}{22.5}$$

$${\rm{CRI}}-{\rm{I}}=\frac{{\rm{{Total}\; {\rm{cholesterol}}}}}{{\rm{HDL}}-{\rm{C}}}$$

An automated hematology analyzer was employed to perform red blood cell (RBC) and white blood cell (WBC) count determinations, as well as to evaluate the mean corpuscular volume (MCV) and the mean platelet volume (MPV). Then, these values were used to calculate the red blood cell width coefficient of variation (RDW-CV) and several inflammatory indexes, namely systemic immune inflammation index (SII), systemic inflammation response index (SIRI), aggregate index of systemic inflammation (AISI), platelet-to-lymphocyte ratio (PLR), monocyte-to-lymphocyte ratio (MLR), and neutrophil-to-lymphocyte ratio (NLR), as follows:

$${\rm{RDW}}-{\rm{CV}}=\frac{{\rm{standard\; deviation\; of\; MCV}}}{{\rm{MCV}}}\times 100$$

$${\rm{SII}}=\frac{{\rm{Neutrophils}}\times {\rm{Platelets}}}{{\rm{Lymphocytes}}}$$

$${\rm{SIRI}}=\frac{{\rm{Neutrophils}}\times {\rm{Monocytes}}}{{\rm{Lymphocytes}}}$$

$${\rm{AISI}}=\frac{{\rm{Neutrophils}}\times {\rm{Monocytes}}\times {\rm{Platelets}}}{{\rm{Lymphocytes}}}$$

$${\rm{PLR}}=\frac{{\rm{Platelets}}}{{\rm{Lymphocytes}}}$$

$${\rm{MLR}}=\frac{{\rm{Monocytes}}}{{\rm{Lymphocytes}}}$$

$${\rm{NLR}}=\frac{{\rm{Neutrophils}}}{{\rm{Lymphocytes}}}$$

### Multi-elemental analysis

Plasma and erythrocyte samples were subjected to multi-elemental analysis according to the method previously developed by González-Domínguez et al. [24]. Briefly, aliquots of plasma (150 µl) and erythrocyte (50 µl) samples were diluted up to 3 ml with an alkaline solution containing 2% 1-butanol (w/v), 0.05% EDTA (w/v), 0.05% Triton X-100 (w/v), and 1% NH_4_OH (w/v). The samples were filtered through 0.45 µm pore size hydrophilic PTFE filters and analyzed using an Agilent 7900 inductively coupled plasma mass spectrometer (Agilent Technologies, Tokyo, Japan). The operating analytical conditions can be found elsewhere [24].

### Statistical analysis

Multi-elemental data were first subjected to quality control [25], and then pre-processed to remove variables containing more than 20% missing values (i.e., erythroid Co and Cr), kNN imputation, log transformation, and Pareto scaling. The adjustment of variables under study to a normal distribution was evaluated through the Kolmogorov–Smirnov test. Then, group comparisons were performed by analysis of variance (ANOVA) followed by the Fisher LSD post hoc test. *p* Values below 0.05 were considered statistically significant. Additionally, linear models with covariate adjustment were employed to control for the influence of the origin of parental obesity (maternal vs. paternal vs. combined) as a potential confounding factor.

## Results

### Anthropometric and biochemical characteristics

As expected, anthropometric measures (i.e., weight, height, BMI) were higher in children with obesity compared to the control group, although no differences were found between PO+ and PO− children (Table 1). The prevalence of family history of type II diabetes was higher among children with obesity, particularly among PO+ subjects, but no differences were observed for other comorbidities (hypertension, dyslipidemia). Children with obesity also exhibited elevated fasting insulin levels and HOMA-IR scores, changes that were more pronounced among subjects with PO. Nevertheless, no differences were found in blood glucose, whereas the obesity-related increment in HbA1c levels was comparable between both study groups. Although the triglyceride content was higher in children with obesity regardless the PO status, subjects in the PO+ group showed a more unfavorable lipid profile, as reflected in lower HDL-C levels and higher CRI-I scores. Childhood obesity was also characterized by increased transferrin levels and TIBC, whereas changes in plasma ferritin, transferrin saturation, and RDW-CV were exclusively significant among PO+ subjects. Finally, children with PO and, to a lesser extent, PO− subjects had increased content of uric acid and lower levels of vitamins (i.e., vitamin D, vitamin B12, folic acid).

**Table 1:** Anthropometric and biochemical characteristics of the study population.

	Control	PO−	PO+	*p* value
Demographic and anthropometric variables				
*N*	30	29	20	
Sex	19♂ 11♀	12♂ 17♀	11♂ 9♀	
Age (years)	8.36 ± 1.41	9.12 ± 1.57	8.92 ± 1.25	NS
Weight (kg)	27.08 ± 4.86	55.16 ± 11.95^a^	59.16 ± 8.25^a^	<0.0001
Weight (*Z*-score)	−0.03 ± 0.69	4.59 ± 2.02^a^	5.67 ± 2.17^a^	<0.0001
Height (cm)	128.12 ± 3.78	141.11 ± 8.93^a^	142.40 ± 4.09^a^	<0.0001
Height (*Z*-score)	−0.10 ± 0.82	1.22 ± 1.13^a^	1.54 ± 1.17^a^	<0.0001
BMI (Kg/m^2^)	16.36 ± 1.27	27.80 ± 4.08^a^	30.47 ± 3.98^a^	<0.0001
BMI (*Z*-score)	−0.36 ± 0.11	4.38 ± 0.33^a^	5.24 ± 0.45^a^	<0.0001
Family history of type II diabetes (%)	18.8	41.4	60.0^a^	0.044
Family history of hypertension (%)	31.2	24.1	25.0	NS
Family history of dyslipidemia (%)	6.2	17.2	15.0	NS
Biochemical variables				
Glucose (mg/dl)	85.52 ± 4.25	82.39 ± 8.22	85.52 ± 4.74	NS
Insulin (µU/ml)	4.50 ± 1.96	12.10 ± 4.60^a^	18.51 ± 6.52^a,b^	<0.0001
HOMA-IR (A.U.)	0.95 ± 0.41	2.71 ± 0.78^a^	3.77 ± 1.41^a^	<0.0001
HbA1c (%)	5.12 ± 0.10	5.34 ± 0.27^a^	5.33 ± 0.20^a^	0.0041
Total cholesterol (mg/dl)	157.37 ± 18.74	161.19 ± 23.99	143.75 ± 23.78	NS
Triglycerides (mg/dl)	39.45 ± 7.81	67.73 ± 18.85^a^	80.17 ± 36.21^a^	<0.0001
HDL-C (mg/dl)	59.12 ± 8.04	48.76 ± 5.40^a^	43.20 ± 2.57^a,b^	<0.0001
LDL-C (mg/dl)	89.00 ± 11.87	93.74 ± 20.28	88.18 ± 21.52	NS
CRI-I (A.U.)	2.72 ± 0.52	3.06 ± 0.41	3.41 ± 0.46^a^	0.0002
Ferritin (ng/ml)	31.26 ± 12.79	35.14 ± 10.40	49.61 ± 11.58^a,b^	<0.0001
Transferrin (mg/dl)	272.64 ± 24.03	304.2 ± 32.39^a^	302.06 ± 27.70^a^	0.014
Transferrin saturation (%)	29.55 ± 9.32	21.11 ± 7.82	15.40 ± 6.08^a^	0.0012
TIBC (µg/dl)	338.00 ± 29.75	383.50 ± 25.16^a^	379.40 ± 32.99^a^	0.0026
RDW-CV (%)	12.27 ± 0.46	12.66 ± 0.48	13.03 ± 0.60^a^	0.0005
Uric acid (mg/dl)	3.51 ± 0.72	4.95 ± 0.57^a^	5.18 ± 0.99^a^	<0.0001
Vitamin D (ng/ml)	32.92 ± 7.29	28.81 ± 7.42	24.72 ± 4.92^a^	0.0032
Vitamin B12 (pg/ml)	556.86 ± 195.33	405.08 ± 141.56	351.45 ± 156.99^a^	0.0142
Folic acid (ng/ml)	8.65 ± 2.97	4.51 ± 1.65^a^	5.24 ± 0.60^a^	0.0004

Values are expressed as the mean ± standard deviation. Superscript letters indicate significant differences between study groups (*p* value < 0.05).

*A.U.* arbitrary units, *BMI* body mass index, *CRI-I* Castelli risk index-I, *HbA1c* glycated hemoglobin, *HDL-C* high-density lipoprotein cholesterol, *HOMA-IR* homeostasis model assessment of insulin resistance, *LDL-C* low-density lipoprotein cholesterol, *NS* non-significant, *PO−* children with obesity without parental obesity, *PO+* children with obesity with parental obesity, *RDW-CV* red blood cell width coefficient of variation, *TIBC* total iron binding capacity.

Superscript letters indicate significant differences between study groups (*p* value < 0.05): ^a^denotes significant difference with respect to the control group, and ^b^denotes significant difference with respect to the PO− group.

### Inflammatory markers

**Fig. 1:**
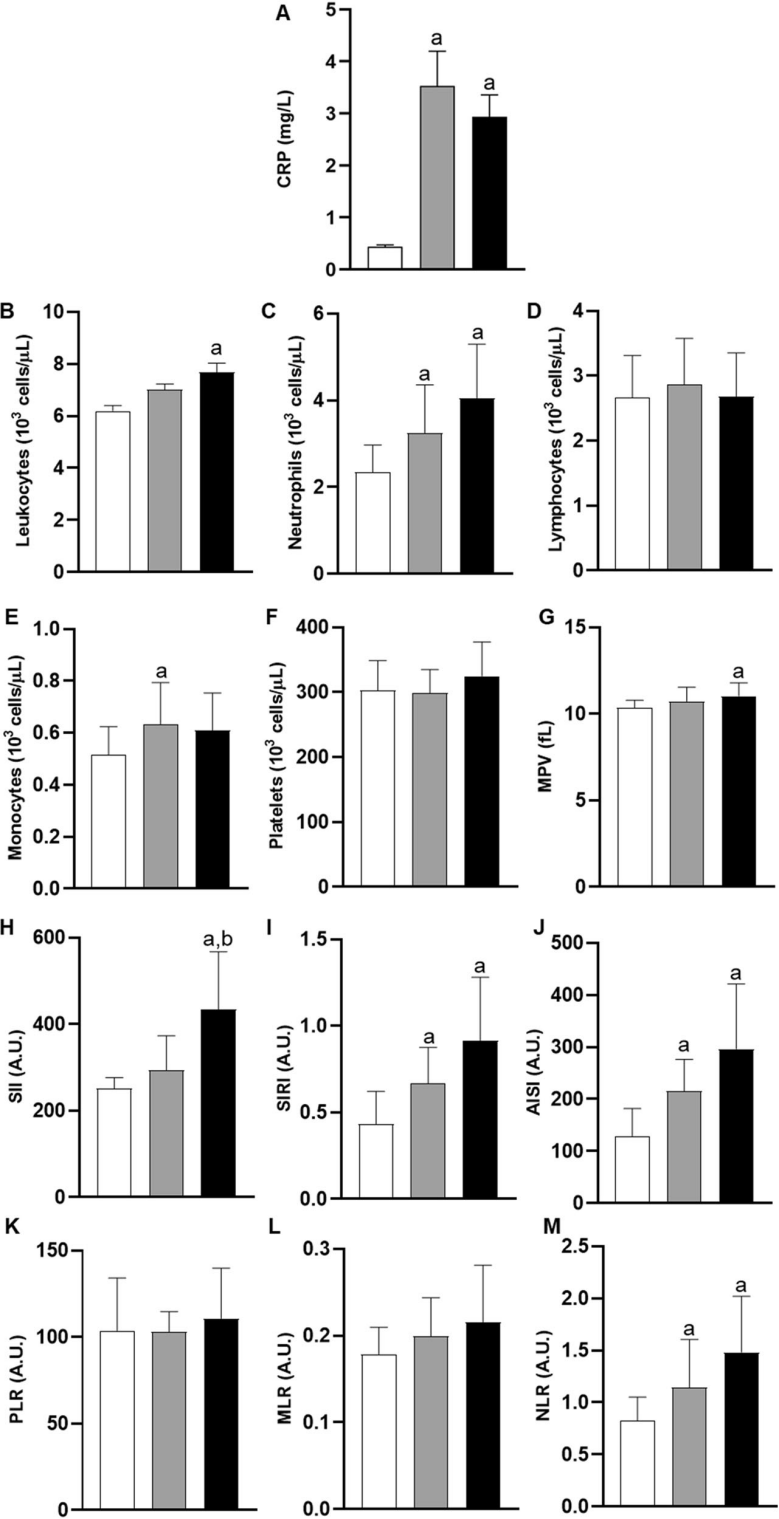
Measurement of inflammation-related variables in the study population. **A** C-reactive protein (CRP) levels, **B** leukocyte count, **C** neutrophil count, **D** lymphocyte count, **E** monocyte count, **F** platelet count, **G** mean platelet volume (MPV), **H** systemic immune-inflammation index (SII), **I** systemic inflammation response index (SIRI), **J** aggregate index of systemic inflammation (AISI), **K** platelet-to-lymphocyte ratio (PLR), **L** monocyte-to-lymphocyte ratio (MLR), **M** neutrophil-to-lymphocyte ratio (NLR). White bars represent the control group, gray bars represent children with obesity without family history of obesity (PO−), and black bars represent children with obesity with family history of obesity (PO+). Values are expressed as the mean ± standard deviation. Letters indicate significant differences between study groups (*p* value < 0.05): (a) denotes significant difference with respect to the control group, and (b) denotes significant difference with respect to the PO− group.

Children with obesity, regardless of family history determinants, had higher blood levels of CRP (Fig. 1A). On the other hand, although both study groups with obesity tended to have higher WBC counts, significant changes in total leukocytes were only observed among individuals with PO when compared with the control group (Fig. 1B). When analyzing specific immune cell populations, the neutrophil count was found to be increased in PO+ and, to a lesser extent, PO− groups (Fig. 1C), whereas the increment in monocytes was only significant in the PO− group (Fig. 1E). On the other hand, PO+ children tended to have higher mean platelet volume (MPV) than healthy control children (Fig. 1G). As a result, WBC-based inflammatory ratios were also found to be raised in childhood obesity, and these differences were more pronounced in PO+ children (Fig. 1H–M).

### Multi-elemental profile

As summarized in Table 2, PO had a significant impact on plasma and erythroid multi-elemental profiles in the offspring. The plasma content of copper was higher in childhood obesity, especially among those subjects with PO, whereas the opposite behavior was observed for zinc and iron. On the other hand, the erythroid content of selenium and iron (and other trace elements, such as copper and zinc, without reaching statistical significance) was found to be higher in PO+ children when compared to the other two study groups. Secondary analyses, using linear modeling and considering the origin of parental obesity (maternal vs. paternal vs. both) as a covariate, provided similar results in the above-mentioned trace elements, thereby suggesting that the influence of parental obesity is independent on which progenitor presents obesity.

**Table 2:** Concentrations of metal and metalloid elements in plasma and erythrocyte samples in the study population.

	Plasma				Erythrocytes			
	Control	PO−	PO+	*p* value	Control	PO−	PO+	*p* value
Arsenic	0.86 ± 1.2	0.95 ± 0.94	0.87 ± 1.4	NS	52.2 ± 23.8	51.7 ± 21.5	46.7 ± 12.2	NS
Cadmium	0.0017 ± 0.0051	0.0018 ± 0.0041	0.0016 ± 0.0044	NS	1.8 ± 2.1	2.2 ± 1.7	1.9 ± 2.1	NS
Chromium	5.8 ± 3.1	5.1 ± 1.8	6.2 ± 2.6	NS	ND	ND	ND	–
Cobalt	1.5 ± 0.4	1.6 ± 0.5	1.5 ± 0.4	NS	ND	ND	ND	–
Copper	1306.6 ± 162.1	1438.8 ± 252.3^a^	1481.3 ± 202.2^a^	0.048	537.7 ± 66.5	562.7 ± 57.9	602.7 ± 155.6	NS
Iron	772.7 ± 172.7	759.2 ± 112.1	637.7 ± 116.8^a,b^	0.043	526,111.1 ± 78,606.4	541,928.6 ± 61,145.2	623,091.4 ± 101,280.5^a,b^	0.025
Lead	0.024 ± 0.0066	0.022 ± 0.0038	0.025 ± 0.0054	NS	61.6 ± 5.5	62.3 ± 6.0	64.6 ± 10.9	NS
Manganese	4.1 ± 1.1	3.8 ± 1.1	3.8 ± 1.2	NS	15.1 ± 9.7	18.7 ± 9.9	13.5 ± 7.5	NS
Molybdenum	2.8 ± 0.6	2.6 ± 0.9	2.9 ± 1.0	NS	23.5 ± 5.2	21.7 ± 4.5	22.1 ± 5.4	NS
Selenium	134.0 ± 15.5	124.4 ± 24.7	127.3 ± 11.0	NS	152.3 ± 27.8	158.8 ± 28.2	198.2 ± 52.7^a,b^	0.016
Zinc	796.7 ± 133.8	714.3 ± 128.1^a^	688.1 ± 107.7^a^	0.041	9212.2 ± 1911.1	9103.9 ± 1262.1	10,172.3 ± 2307.7	NS

Values are expressed as the mean ± standard deviation (µg/l).

*ND* non-detected, *NS* non-significant, *PO−* children with obesity without parental obesity, *PO+* children with obesity with parental obesity.

Superscript letters indicate significant differences between study groups (*p* value < 0.05): ^a^denotes significant difference with respect to the control group, and ^b^denotes significant difference with respect to the PO− group.

## Discussion

Although family history of obesity is known to increase the susceptibility of developing obesity in the infant, its influence in concomitant metabolic complications has scarcely been investigated. Interestingly, we found here for the first time that children with obesity and PO display exacerbated disturbances in multiple intertwined obesity-related pathogenic events. On the one hand, children in the PO+ group had higher blood insulin levels at fasting and reduced insulin sensitivity (i.e., increased HOMA-IR scores). This concurs with the results by Martínez-Villanueva et al., who reported that children with at least one parent with obesity (either the mother, the father, or both) present more pronounced impairments in carbohydrate metabolism, as mirrored in increased levels of insulin and glycated hemoglobin, higher HOMA-IR scores, and lower WBISI (whole-body insulin sensitivity index) scores [26]. In this respect, other studies conducted in rodent models have described that both maternal and paternal obesity may affect glucose homeostasis, insulin sensitivity, and the lifetime of pancreatic islets in the offspring [27, 28], thereby predisposing to insulin resistance and type 2 diabetes [10]. Furthermore, these children also showed an unhealthier lipid profile, as reflected in increased TG levels, decreased HDL-C, and higher CRI-I scores (Table 1), in agreement with other authors repeatedly describing a positive association between parental obesity and various adiposity markers in children [29–31]. In this line, Ornellas et al. reported that diet-induced PO significantly worsen lipid metabolism in the progeny by triggering the activation of hepatic lipogenesis and the impairment of β-oxidation (this latter exclusively among the offspring of mothers with obesity) [32]. These dyslipidemia factors were in turn accompanied by lower blood levels of vitamins D and B12, whose deficiency has been associated with higher risk of developing obesity-related comorbidities [33]. As expected, since fat accumulation is known to provoke chronic inflammation through different mechanisms (e.g., cytokine secretion, immune response activation), childhood obesity was also characterized by a pro-inflammatory status mirrored in increased WBC counts, inflammatory indexes, as well as CRP and uric acid levels. Despite both obesity study groups had similar distributions in anthropometric variables, children with PO interestingly exhibited a more pronounced raise in these inflammatory markers compared to their PO− counterparts. Supporting these results, a pioneer study conducted in high-fat diet fed mice proved that parental and grandparental obesity may affect systemic immune changes in the offspring, as indicated by adipose tissue fibrosis, macrophage infiltration, as well as increased levels of pro-inflammatory cytokines (IL-1β) and decreased levels of anti-inflammatory cytokines (IL-10) [34]. In another study, combined paternal and maternal obesity was found to augment hypothalamus inflammation in the progeny, which might be behind a disturbed regulation of mechanisms controlling appetite (i.e., leptin signaling, Janus kinase/signal transducers and activators of transcription), thereby contributing to the onset of hyperphagia and obesity in the long term [35]. Although similar results have not yet been replicated in human populations, family history of diabetes has been associated with higher subclinical inflammation among children [36], in line with our results.

The above-mentioned exacerbation in typical metabolic complications accompanying childhood obesity were also reflected in a perturbed multi-elemental profile, thereby supporting a pivotal role of trace elements in their modulation. Multi-elemental analysis revealed a significant increase in plasmatic levels of copper, together with decreased zinc content, among children with obesity (more pronounced within the PO+ group without reaching statistical significance). As recently reported, this observation is likely to be the result of the well-described antagonistic interactions between these two essential elements [17, 20]. Under a pro-inflammatory status, cytokines promote the expression of zinc transporters and induce intracellular copper efflux into the circulation, which is finally mirrored in increased Cu/Zn ratios. In turn, this increment of copper content in circulation contributes to alter lipid metabolism and to exacerbate oxidative stress by generating free radical species [37, 38], whereas the depletion of zinc metalloproteins, which participate in insulin production, storage, and action [39], might be partially responsible for the alterations described above in insulin homeostasis. Similarly, inflammation also modulates iron metabolism by inducing hepcidin expression and by inhibiting intestinal iron absorption and its efflux from iron-transporting tissues [40], which results in increased accumulation of ferritin-bound iron in cellular cytoplasm and decreased circulating concentrations, in line with our findings. This inflammatory-mediated expression of metal transporters could be in turn responsible for the accumulation of various trace elements (i.e., iron, selenium, copper, zinc) within the erythrocyte of PO+ children, in line with recent studies [17, 20]. In this venue, iron sequestration in pancreatic β cells and adipocytes has been reported to trigger the formation of radical oxygen species, subsequently provoking impairments in insulin secretion and sensitivity [41, 42]. Noteworthy, this circulating iron deficiency can be regarded as an expression of the anemia of inflammation that frequently occurs in obesity. Concurring with this data, we also observed increased plasma levels of ferritin and transferrin, higher TIBC, decreased transferrin saturation, and elevated RDW-CV values, which are considered typical hallmarks of obesity-related anemic states [43, 44]. Altogether, we hypothesize that the exacerbated pro-inflammatory environment in PO+ subjects (Fig. 1) could be behind aggravated impairments in iron metabolism leading to anemia.

This study is strengthened by the use of a population-based cohort exclusively consisting of prepubertal children, which allows minimizing inter-individual variability factors allocated to pubertal development and, therefore, to identify strong associations between PO and metabolic alterations in children. Thus, the comprehensive characterization of typical obesity-related pathogenic events (i.e., abnormal insulin homeostasis, dyslipidemia, inflammation), combined with the application of high throughput multi-elemental approaches, enabled us to decipher the molecular imprint of parental obesity. On the other hand, the main limitation of this work is the inherent lowering in statistical power that the stratification of the study population entails (i.e., PO+ vs. PO−). Furthermore, it would also be interesting to separately address the influence of maternal and paternal obesity in the development of obesity-related complications in the offspring. This makes future studies in larger and independent cohorts mandatory to validate our findings.

## Conclusions

Herein, we have demonstrated for the first time the relevance of family history of obesity as a potential risk factor in the development of obesity-related metabolic complications in the offspring. Parental obesity has been found to associate with disturbances in trace elements (i.e., copper, zinc, iron, selenium), which could predispose to exacerbate related comorbidities, such as altered insulin homeostasis, dyslipidemia, and inflammation. Therefore, this study highlights the crucial importance of implementing preventive measures even before birth to avoid future complications during childhood.

## Data Availability

The datasets generated during and/or analyzed during the current study are available from the corresponding author on reasonable request.
